# Virtual screening for potential inhibitors of β(1,3)-D-glucan synthase as drug candidates against fungal cell wall

**DOI:** 10.1080/21556660.2020.1734010

**Published:** 2020-03-11

**Authors:** Zinat Farhadi, Tayebeh Farhadi, Seyed MohammadReza Hashemian

**Affiliations:** a Chronic Respiratory Diseases Research Center (CRDRC), National Research Institute of Tuberculosis and Lung Diseases (NRITLD), Shahid Beheshti University of Medical Sciences, Tehran, Iran; b Behavioral Disease Counseling Center, Marvdasht Health Center, Shiraz University of Medical Sciences, Shiraz, Iran; c Department of Microbiology, Shiraz Branch, Islamic Azad University, Shiraz, Iran; d Critical Care Department, Farhikhtegan Hospital, Tehran Medical Branch, Islamic Azad University, Tehran, Iran

**Keywords:** Candidiasis, caspofungin, β(1,3)-D-glucan synthase, oral drug, virtual screening

## Abstract

**Background:**

To enhance the outcome in patients with invasive candidiasis, initiation of an efficient antifungal treatment in a suitable dosage is necessary. Echinocandins (e.g. caspofungin) inhibit the enzyme β(1,3)-D-glucan synthase of the fungal cell wall. Compared to azoles and other antifungal agents, echinocandins have lower adverse effects and toxicity in humans. Echinocandins are available in injectable (intravenous) form.

**Methods:**

In this study, to identify the novel oral drug-like compounds that affect the fungal cell wall, downloaded oral drug-like compounds from the ZINC database were processed with a virtual screening procedure. The docking free energies were calculated and compared with the known inhibitor caspofungin. Four molecules were selected as the most potent ligands and subjected to hydrogen bonds analysis.

**Results:**

Considering the hydrogen bond analysis, two compounds (ZINC71336662 and ZINC40910772) were predicted to better interact with the active site of β(1,3)-D-glucan synthase compared with caspofungin.

**Conclusion:**

The introduced compound in this study may be valuable to analyze experimentally as a novel oral drug candidate targeting fungal cell walls.

## Introduction

Invasive candidiasis has been considered a significant reason of death in immuno-compromised and critically ill patients^1–6^. Invasive candidiasis can initially be managed in critically ill patients by utilizing an echinocandin, e.g. caspofungin^7^^,^^8^. Caspofungin inhibits the enzyme β(1,3)-D-glucan synthase of the fungal cell wall in a non-competitive manner leading to inhibition of the synthesis of β(1,3)-D-glucan. β(1,3)-D-glucan is a crucial element of the cell wall of many fungal species and forms a solid three-dimensional matrix that is involved in shape determination and mechanical strength of the cell wall^9^^,^^10^.

Inhibiting the synthesis of β(1,3)-D-glucan has both fungistatic and fungicidal outcomes. Impediment of the cell wall synthesis limits the cell growth and has a fungistatic outcome. An alternation in the integrity of the fungal cell wall causes losing the mechanical strength of the cell wall, resulting in a deficiency of the cell to maintain the intracellular osmotic pressure and destruction of the fungal cell (fungicidal effect)^9^^,^^11–13^.

Echinocandins are weakly bioavailable for oral administration because of their large molecular weight; hence, they were approved only for intravenous use. IV injection of drugs may cause local reactions, requires a functioning cannula, and is more expensive and labor intensive than other routes. Cannulation is distressing to some patients and cannulae are prone to infection. Compared to intravenous route of drug administration, oral administration of drugs has some advantages. They are easy for use, cost-effective, preferred by patients, and expand the duration of action because of slow release^14–16^.

In ICU patients that do not suffer from sepsis, patients who are transferred from the ICU to the ward and discharged patients from the hospital, oral antifungal drugs (azoles) are usually administrated as an alternative to injectable form. Azoles disrupt the ergosterol in the fungal cell membrane. Both fungi and humans are eukaryotes and have a similar cell membrane structure. For this reason, the use of oral drugs acting on the fungal cell membrane can cause side-effects and toxicity in humans. Echinocandins destroy the cell wall of fungi. Therefore, they have lower adverse effects and toxicity than azoles and other antifungal agents. Echinocandins are available in the injectable (intravenous) form, hence, discovering novel drug candidates in oral form and capable of destroying the fungal cell wall is necessary.

Some intrinsic properties of drug candidates including absorption, distribution, metabolism, and excretion (ADME) have a significant effect on the chance of usefulness of a therapeutic agent in the human body^14^^,^^15^. According to Lipinski’s rule of five, a compound should have some standards criteria including the number of hydrogen bond donors (not more than five), the number of hydrogen bond acceptors (not more than 10), the molecular weight (not higher than 500 Daltons) and the logP value (not greater than five) to be considered an orally active drug candidate^14^. Lipinski’s rule of five is a rule that describes molecular characteristics significant for a drug’s pharmacokinetics in the human body, including their absorption, distribution, metabolism, and excretion (ADME)^14^.

Virtual screening is a computer-aided procedure that is extensively employed in drug discovery tasks. By virtual screening, one can examine the potency of thousands to millions of agents to be active against a target system^17^^,^^18^. In the structural-based virtual screening, the geometries of interactions and binding affinities between a library of ligands and a specific receptor is predicted by using ligand-receptor docking analysis^19^. In many attempts for computational drug discovery, the aim was to find novel leads for protein receptors^20–27^. Molecular docking analysis is a suitable technique to evaluate the binding mode of ligands to their targets and has been effectively used in a lot of applications^28–31^.

In this study, it was an attempt to virtual screening of a library of orally active drug-like compounds to detect potential lead inhibitors of of β(1,3)-D-glucan synthase of the fungal cell wall, which bind more effectively than caspofungin through scoring and ranking. The oral drug-like leads that were introduced in this study may be hopeful candidates as efficient oral drugs against fungal infections.

## Methods

### Retrieval of ligands

In this study, a Windows 7 operating system with a seven-core processor was used for the computational studies. In order to retrieve oral active drug candidates to inhibit β(1,3)-D-glucan synthase, the intrinsic properties, including molecular weight, xlogP range, net charge range, hydrogen donors range, and hydrogen acceptors range were used as virtual screening criteria. The “property search” tool of the ZINC database (http://zinc.docking.org) was employed to achieve an appropriate number of orally active drug-like molecules. The molecular weight (g/mol) in a range of 240–290, xlogP in a range of −4 to −2, net charge in a range of −2 to 0, rotatable bonds in a range of 0–5, polar surface area (A° 2) in a range of 120–150, hydrogen donors in a range of 0–4, hydrogen acceptors in a range of 5–10, polar desolvation (kcal/mol) in a range of −70 to −40, and apolar desolvation (kcal/mol) in a range of −2 to 9 were submitted to the “property search” tool as queries. A dataset containing 2,000 drug-like compounds (in MOL format) was gained as output. OpenBabel tool in PyRx 0.8 software was used to change the format to pdbqt^32^. The obtained molecules were considered ligands and subjected to the virtual screening process.

### Tertiary structure prediction

The sequence of the 1,3-β-D-glucan synthase protein (as receptor) was obtained from UniProtKB (ID: A2QLK4). I-TASSER (http://zhanglab.ccmb.med.umich.edu/I-TASSER) was employed to predict the tertiary structure of the receptor. I-TASSER utilizes a hierarchical modeling procedure based on multiple threading alignments. I-TASSER modeling initiates from the PDB structure templates recognized by the LOMETS program. Only the highest significance templates are selected by I-TASSER. The ten best templates selected from the LOMETS threading programs are shown in the output page of the I-TASSER. Usually, one template of the highest *Z*-score is selected from each threading program^33^^,^^34^.

Overall, homology modeling, threading, and *ab initio* methods are available for protein modeling. In this study, as was mentioned above, the LOMETS threading program in I-TASSER was used to model the receptor. Recently, in the field of antifungal drug discovery, the tertiary structure of other antifungal targets has been predicted using the homology modeling method^35^^,^^36^.

By energy minimization of the modeled receptor, it was attempted to relieve the model from the bad contacts. In a stable system, atoms are arranged with minimal energy. Therefore, the stability of a model protein can be improved by fixing distorted geometries of the system *via* energy minimization^37^. The energy of the modeled receptor was minimized by using Gromos96 force field^38^, implemented in Swiss-PdbViewer v.4.2^39^.

### Model evaluation

Ramachandran plot was obtained from MolProbity (http://molprobity.biochem.duke.edu/) to examine the model of 1,3-β-D-glucan synthase before and after energy minimization. Ramachandran plot checks the residue-by-residue stereo-chemical quality of modeled constructs for their structural quality and reliability.

### Active site

**Figure 1.**
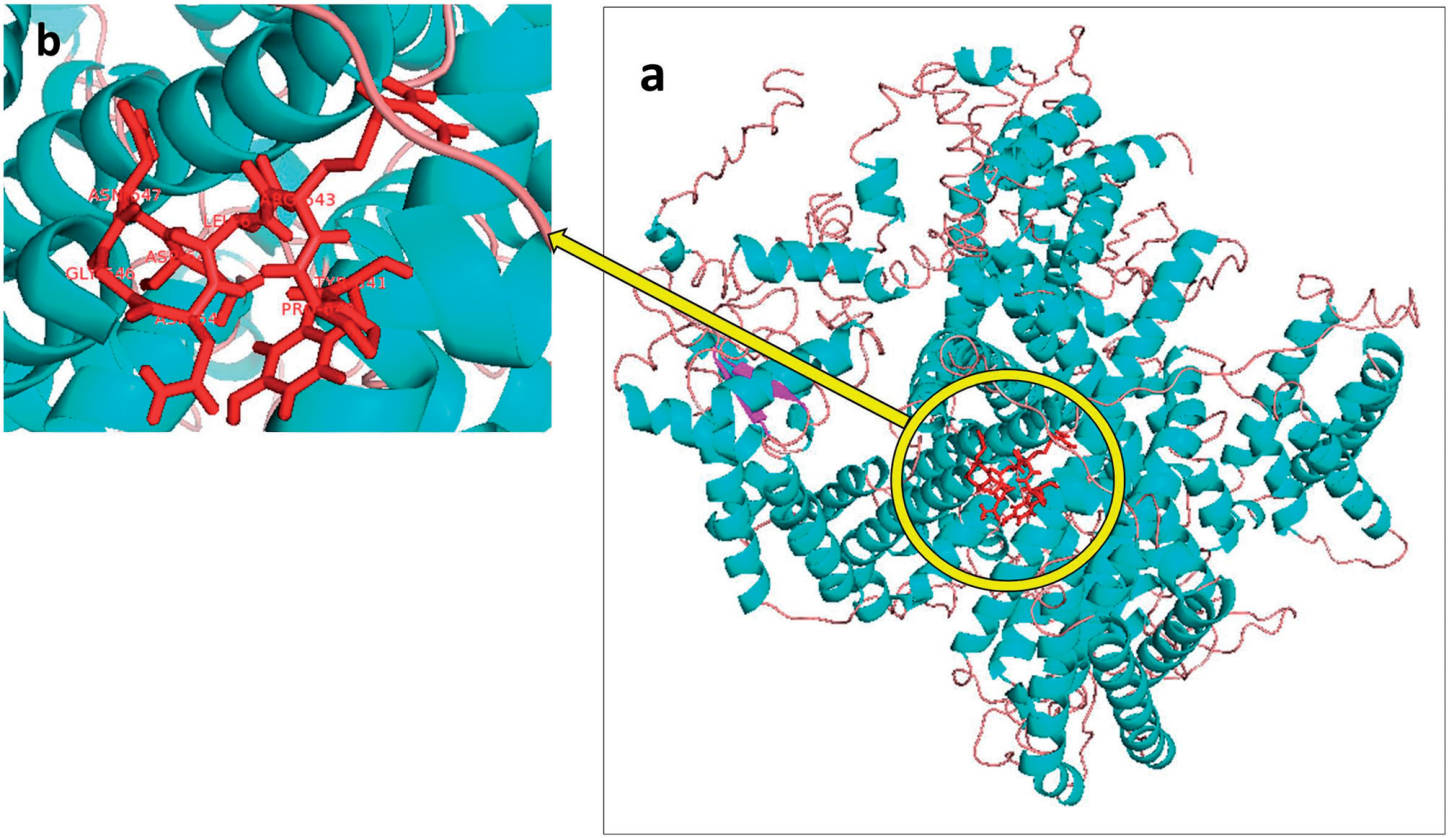
(a) 3D structure of 1,3-β-D-glucan synthase, (b) the 8-amino-acid portion (AA-641 to AA-648) of the 1,3-glucan synthase that is proposed as the drug binding site of the receptor.

Coordinates of a ligand (inhibitor) in a target protein grid have been considered as an active binding site of the target. In the sequence of 1,3-β-D-glucan synthase, there is a predicted 89-amino-acid-domain (AA-584 to AA-673) containing an 8-amino-acid portion^40^^,^^41^. The 8-amino-acid portion (AA-641 to AA-648) has been mapped in some topology models to the cytoplasmic face of the plasma membrane and has been proposed as the echinocandin binding site of 1,3-glucan synthase (Figure 1(b))^40^^,^^41^. Accordingly, in this study, the 8-amino-acid portion (Tyr-641, Pro-642, Arg-643, Leu-644, Asn-645, Gly-646, Asn-647, Asp-648) of the receptor (Figure 1(b)) was used as the ligand binding site.

### Virtual screening

Virtual Screening of compound libraries against desired and potential receptors (targets) can be carried out using Pyrx 0.8 software^32^. Here, by using AutoDock Vina module in PyRx 0.8^42^, all drug-like agents retrieved from ZINC were subjected to docking analysis against the active site of the receptor. The X, Y, and Z dimensions of the grid map for docking calculations were set to 16.27, 16.06, and 15.36 Å, respectively. The grid map was centered on the ligand binding site of the receptor. The X, Y, and Z centers of the grid map were set to 105.80, 88.81, and 73.41, respectively.

Overall, in a respective docking complex, low binding energy between the active site of a receptor and ligand may indicate that the receptor is docked with its ligand in a favorable manner. The results of the docking analysis may show the appropriate binding interaction and therefore the possible activity of the searched drug candidates^21–27^.

In this study, the binding affinity of the receptor active site with each ligand was estimated based on free energy of interactions. Docking of 1,3-β-D-glucan synthase and caspofungin was performed as control. The free energy of interaction between the receptor and the original drug (caspofungin) were calculated and compared with the energy between the receptor and the obtained compound library. The molecules that had considerable lower free energies compared to caspofungin were considered “lead molecules” and utilized for hydrogen bond analysis.

The number of the hydrogen bonds between the receptor and caspofungin were calculated and compared with the hydrogen bonds between the receptor and the lead molecules. The leads that had more hydrogen bonds than caspofungin were considered to have better binding affinity to the receptor and consequently be hopeful drug candidates against fungal infections for further evaluations.

## Results

### Tertiary structure prediction

The 3D structure of 1,3-β-D-glucan synthase was modeled by using I-TASSER. The top seven threading templates used by I-TASSER were as follows: 5x0m, 3jbr, 5xsy, 6c96, 6nq0, 6edo, and 4ai6. Model 1 was selected as the best model based on the C-score (−1.88) (Figure 1). To determine the quality of I-TASSER predicted models, C-score may be used. The C-score is usually in a range of −5 to 2, and a greater value of C-score shows higher validity of a predicted model.

By using the appropriate software, the energy of the model 1 was minimized to relieve the model from bad contacts. The total energy of the minimized model was 6899.6 KJ/mol.

### Model evaluation

**Figure 2.**
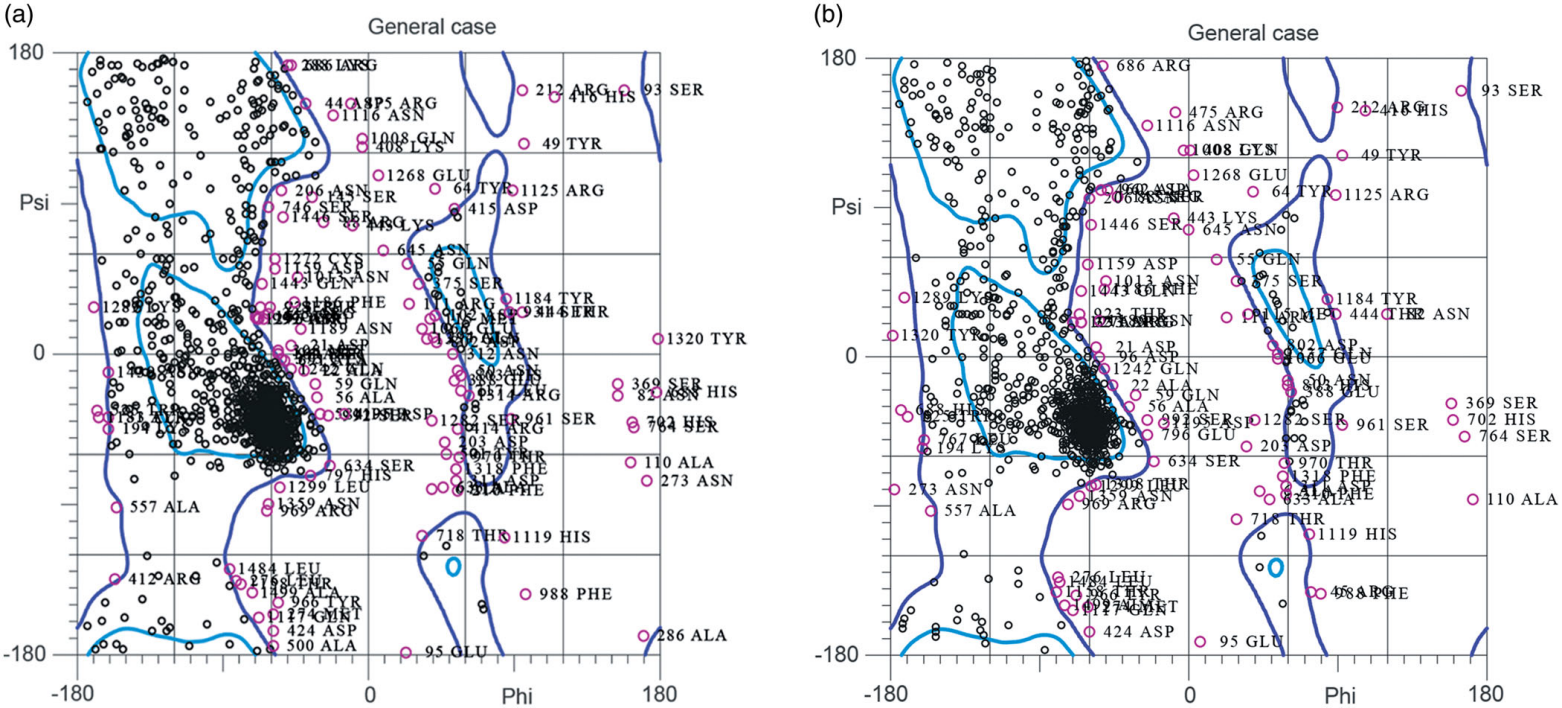
(a) Ramachandran plot for the model of 1,3-β-D-glucan synthase before minimization, (b) Ramachandran plot for the energy minimized model of 1,3-β-D-glucan synthase.

The results of the model evaluation before energy minimization were as follows: the phi/psi angles of 69.23% and 12.48% of the residues were located in the favored regions and Ramachandran outliers, respectively. A Ramachandran plot for the model of 1,3-β-D-glucan synthase before energy minimization is represented in Figure 2(a).

The results of the model evaluation after energy minimization were as follows: the phi/psi angles of 72.56% and 9.95% of the residues were located in the favored regions and Ramachandran outliers, respectively. A Ramachandran plot for the energy minimized model of 1,3-β-D-glucan synthase is represented in Figure 2(b).

### Docking analysis of 1,3-β-D-glucan synthase with caspofungin

**Figure 3.**
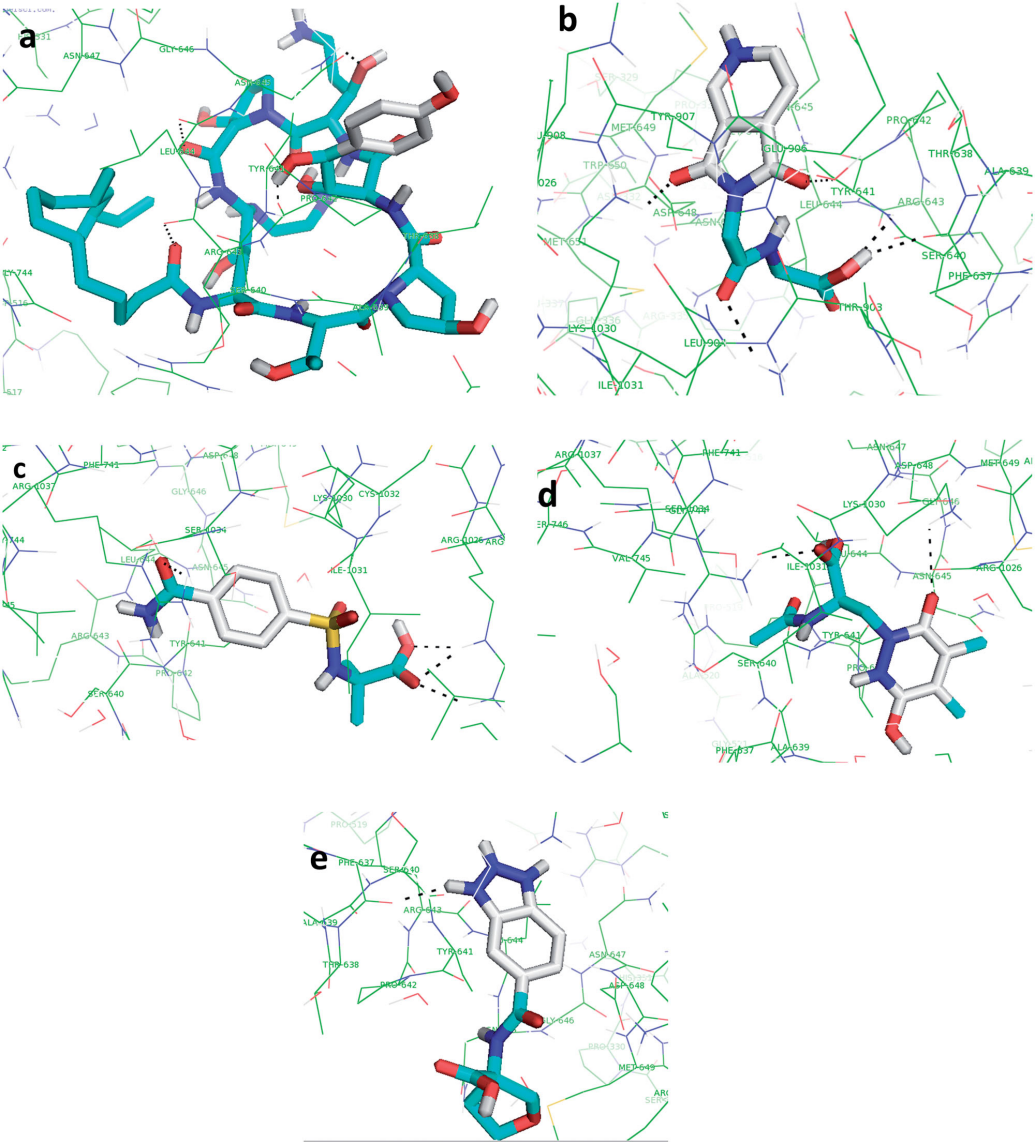
The interactions of the caspofungin (a), lead 1 (b), lead 2 (c), lead 3 (d), and lead 5 (e) with the receptor. Hydrogen bonds between each ligand and the receptor are shown as dotted black lines.

Results of docking analysis between 1,3-β-D-glucan synthase and caspofungin (Figure 3(a)) represented binding energy of −5.3 kcal/mol for the caspofungin-receptor complex. Caspofungin forms four hydrogen bonds with the active site residues: one each with Pro642, Arg643, Leu644, and Asn645 (Figure 3(a)).

### Virtual screening

Virtual screening of 2000 drug-like compounds retrieved from the ZINC database against 1,3-β-D-glucan synthase was done *via* AutoDock Vina in PyRx. The software produced nine different poses for each ligand based on the receptor–ligand binding energies. The best scoring pose was selected for each ligand.

Among all retrieved compounds, compounds that had a binding energy lower than −7.00 kcal/mol in a docking complex with the receptor were selected as the best ranked molecules. The molecules ZINC71336662, ZINC40910772, ZINC81131432, and ZINC85849956 presented a binding energy of −7.2, −7.1, −7.0, and −7.0 kcal/mol, respectively (Table 1).

**Table 1.** Comparison docking results of the four novel inhibitors of 1,3-β-D-glucan synthase (as lead compounds) along with the known inhibitor (caspofungin).

IDs	ZINC ID	Binding energy (kcal/mol)	Interacting residues–No. of H-bond (s)
Lead 1	ZINC71336662	−7.2	Phe637-1 Tyr907-1 Tyr641-1 Lys1030-1 Arg1037-1
Lead 2	ZINC40910772	−7.1	Arg1026-3 Arg1037-1
Lead 3	ZINC81131432	−7.0	Arg1037-1 Lys1030-1
Lead 4	ZINC85849956	−7.0	Phe637-1
Caspofungin	—	−5.3	Pro642-1 Arg643-1 Leu644-1 Asn645-1

The minimum binding energy of the mentioned ligands signified that the receptor was satisfactorily docked with them. Moreover, the lower binding energy of the leads compared to caspofungin in respective docking complexes explains that they may bind competitively into the binding site of the target compared to caspofungin. The introduced lead molecules in this study may be considered valuable lead agents to subject experimental analysis. The compounds ZINC71336662, ZINC40910772, ZINC81131432, and ZINC85849956 were ranked based on the binding energies and named Lead 1 to 4, respectively (Table 1).

Each lead molecule was analyzed in terms of the number of hydrogen bonds that it can form with the active site of the receptor. Docking results of the leads with the receptor along with the identified inhibitor (caspofungin) are shown in Table 1. Considering Table 1, lead 1 forms five hydrogen bonds with the ligand binding site, and leads 2, 3, and 4 interact with the target by four, two, and one hydrogen bonds, respectively. Table 2 represents the name, chemical structure, and molecular weight of the novel inhibitors along with the control (caspofungin). The hydrogen bonds of the leads 1–4 with the receptor are displayed in Figures 3(b–e), respectively.

**Table 2.** The name, molecular weight, and chemical structure of the four best lead molecules and the known inhibitor caspofungin.

ID	SMILES	Molecular weight (g/mol)	Chemical structure
Lead 1	O=C(O)CNC(=O)CN1C(=O)c2ccncc2C1=O	263.209	
Lead 2	CC(C)(NS(=O)(=O)c1ccc(C(N)=O)cc1)C(=O)O	286.309	
Lead 3	CC(=O)N[C@H](Cn1[nH]c(=O)c(C)c(C)c1=O)C(=O)O	269.257	
Lead 4	O=C(N[C@@]1(C(=O)O)CCOC1)c1ccc2[nH]nnc2c1	276.252	
Caspofungin	CCC(C)CC(C)CCCCCCCCC(=O)N[C@ H]1C[C@H] ([C@H](NC(=O)[C@@H]2[C@H](CCN2C(=O)[C@@H] (NC(=O)[C@@H](NC(=O)[C@@H]3C[C@H](CN3C(=O)[C@ @H] (NC1= O)[C@@H](C)O)O)[C@@H]([C@H](C4=CC=C(C= C4)O)O)O)[C@@H](CCN) O)O)NCCN)O	1093.3	

Lead 1 (Figure 3(b)) showd a binding energy of −7.2 kcal/mol, which is much higher than the binding energy of receptor and caspofngin (−5.3 kcal/mol). Lead 1 formed five hydrogen bonds with Phe637 (one H-bond), Tyr907 (one H-bond), Tyr641 (one H-bond), Lys1030 (one H-bond), and Arg1037 (one H-bond) (Figure 3(b)). Lead 2 (Figure 3(c)) shows a binding energy of −7.1 kcal/mol. Lead 2 formed four hydrogen bonds with Arg1026 (three H-bond) and Arg1037 (one H-bond) (Figure 3(c)). Lead 3 (Figure 3(d)) shows a binding energy of −7.0 kcal/mol. Lead 3 formed two hydrogen bonds with Arg1037 (one H-bond) and Lys1030 (one H-bond) (Figure 3(d)). Lead 4 (Figure 3(e)) shows a binding energy of −7.0 kcal/mol. Lead 4 formed one hydrogen bond with Phe637 (one H-bond) (Figure 3(e)).

Considering the results of hydrogen bond analysis, leads 1 and 2 form more hydrogen bonds with the receptor compared to caspofungin and they may be the most hopeful lead molecules. They can be powerful drug candidates for competitive inhibition of 1,3-β-D-glucan synthase.

## Discussion

Virtual screening technology has been considered a helpful technique for discovering novel therapeutic candidates. Virtual screening has some advantages such as reliability and cost and time effectiveness. In structure-based virtual screening method, a collection of drug candidate molecules are screened against a macromolecular target of interest and the binding affinity of the drug candidates to the target is predicted. By using such a strategy, among a large number of molecules, the molecules that have higher binding affinity for a desired receptor can be selected as lead drug candidates (leads) for more evaluation^43^^,^^44^.

In order to select the leads as hopeful drug candidates, “binding energy” between each molecule (ligand) and the receptor can be a suitable criterion and calculated through docking analysis^20^. Overall, evaluation of some factors such as “binding energy” and “number of hydrogen bonds” between ligands and their receptors has been interpreted as a computational filtering method to limit the number of drug candidates for experimental analysis^16^. The degree of ligand-receptor binding refers to the binding affinity. The energy released due to the bond formation or, rather, interaction of the ligand and protein is termed in the form of binding energy. The free energy of a favorable reaction is negative. Lesser the binding energy, the better is the binding of the ligand and protein^16^^,^^21–27^. In this study, the virtual screening software was employed to screen a library of agents against the protein receptor, 1,3-β-D-glucan synthase. The results of this study may be promising in the field of discovering and designing novel drug candidates against fungal infections.

The docking method is usually used to find novel ligands for a desired receptor. In high-throughput and structure-based virtual screenings of small molecules (as drug candidates) the method has been widely used^16^.

Despite huge developments and successful employments of docking analysis in drug designing tasks, some challenges remain unanswered yet. A challenge is because of simplified techniques employed in high-throughput screening of ligands to heighten the speed of checking thousands to millions of compounds^20^. Another challenge is the assumption that similar ligands bind to the target in the same mode. This issue may reduce the size of library and lead us to ignore some valuable drug candidates with more rigid shape complementarities in ligand-receptor complexes^45^. Unavailable X-ray crystallography structures of protein receptors and limitations to determine the structure of receptors experimentally are other challenges^16^^,^^46^. The probability to have a successful virtual screening procedure can be high when researchers are aware of limitations and estimations of docking analysis.

Detection, evaluation, and development of novel efficient and safe drug candidates with a sufficient inhibitory function against pathogens are essential in the pharmaceutical industry. Overall, agents that are able to block the ligand binding sites of a receptor can be considered interesting potential inhibitors against the desirable receptor^47^. Hence, the introduced lead molecules in this study may be evaluable candidates for investigation through wet-lab analysis and could be potential inhibitors against 1,3-β-D-glucan synthase. The activity as well as cytotoxicity of the lead molecules can be relieved through *in vitro* and *in vivo* analysis.

## Transparency

### Declaration of financial/other relationships

The authors and peer reviewers on this manuscript have no relevant financial or other relationships to disclose.
